# Dose-specific effect of simvastatin on hypoxia-induced HIF-1α and BACE expression in Alzheimer’s disease cybrid cells

**DOI:** 10.1186/s12883-015-0390-5

**Published:** 2015-07-31

**Authors:** Jin-Heon Jeong, Kyu Sun Yum, Jun Young Chang, Manho Kim, Jin-young Ahn, SangYun Kim, Paul A Lapchak, Moon-Ku Han

**Affiliations:** Department of Neurology, College of Medicine, Chungbuk National University, Chungbuk National University Hospital, Cheongju, Korea; Department of Neurology, College of Medicine, Konyang University, Konyang University Hospital, Daejeon, Korea; Department of Neurology, College of Medicine, Seoul National University, Seoul National University Bundang Hospital, Seongnam, Korea; Department of Neurology, College of Medicine, Seoul National University, Seoul National University Hospital, Seoul, Korea; Department of Neurology, Seoul Medical Center, Seoul, Korea; Departments of Neurology and Neurosurgery, Cedars-Sinai Medical Center, Los Angeles, USA

**Keywords:** Alzheimer’s disease, Cybrid cell, Statin, Hypoxia, HIF-1α, BACE

## Abstract

**Background:**

Alzheimer’s disease (AD) is associated with vascular risk factors; brain ischemia facilitates the pathogenesis of AD. Recent studies have suggested that the reduction of AD risk with statin was achieved by decreased amyloidogenic amyloid precursor protein.

**Methods:**

We used mitochondrial transgenic neuronal cell (cybrid) models to investigate changes in the levels of intracellular hypoxia inducible factor 1α (HIF-1α) and β-site amyloid precursor protein cleaving enzyme (BACE) in the presence of simvastatin. Sporadic AD (SAD) and age-matched control (CTL) cybrids were exposed to 2 % O_2_ and incubated with 1 μM or 10 μM simvastatin.

**Results:**

There was no significant difference between cell survival by 1 or 10 μM simvastatin in both SAD and CTL cybrids. In the presence of 1 μM simvastatin, intracellular levels of HIF-1α and BACE decreased by 40–70 % in SAD, but not CTL cybrids. However, 10 μM simvastatin increased HIF-1α and BACE expression in both cybrid models.

**Conclusion:**

Our results suggest demonstrate differential dose-dependent effects of simvastatin on HIF-1α and BACE in cultured Alzheimer’s disease cybrid cells.

## Background

Alzheimer’s disease (AD) is a progressive neurodegenerative disorder that affects memory function; it is characterized by the formation of senile plaques composed of beta amyloid (Aβ) [1]. Vascular risk factors such as hypertension and diabetes mellitus have an established association with AD, and over 30 % of AD patients show evidence of cerebral infarcts [2, 3]. Brain ischemia contributes to the pathogenesis of AD [2, 3], and the molecular link between hypoxia and Aβ production is well established. Hypoxia increases expression of β-site amyloid precursor protein cleaving enzyme (BACE) via induction of hypoxia inducible factor 1α (HIF-1α), resulting in increased β-secretase activity and Aβ production [4–6].

Statins (HMG-CoA reductase inhibitor) have some utility in stroke prevention and studies have shown that statin administration can reduce the incidence of and improve functional outcomes after ischemic stroke [7]. The neuroprotective properties of statins have been demonstrated in models of cerebral ischemia [8]. Beyond their originally defined role in lowering cholesterol, statins have been used to manage neurodegenerative disorders such as vascular dementia and AD [9], because they can improve vascular integrity. Statins also alter HIF-1α related gene expression by modulating DNA-binding activity [10]; HIF-1α is essential to the cellular and systemic response to hypoxia [11]. Epidemiologists have found up to a 70 % decreased risk of AD in people taking statins [12] and several studies have shown that statins reduce the production of Aβ [13, 14]. The effects of statin differed according to dose; low-dose simvastatin decreases Aβ production without increment of Aβ release [15].

Cytoplasmic hybrid (cybrid) cell models have been used to demonstrate the role of dysfunctional mitochondria in AD pathogenesis. Studies using this technique have shown that Sporadic AD (SAD) cybrids have increased intracellular and/or extracellular Aβ levels that induce apoptotic neuronal death [16]. SAD cybrids also show increased accumulation of oxidative stress markers such as trans-4-hydroxy-2-nonenal adducts [17], which play an integral role in cellular toxicity. Cybrids are thus a good model for the study of mechanisms involved in cellular pathology. We used cybrids to investigate the changes in intracellular HIF-1α and BACE levels in the presence of simvastatin under hypoxic conditions.

## Methods

### Cell culture experiments

Mitochondrial transgenic neuronal cells (cybrids) of SAD and age-matched controls (CTL) were used to investigate the effect of simvastatin on HIF-1α and BACE expression under hypoxic conditions. We used established Alzheimer’s disease cybrid models that were created by transferring mitochondria from a living AD patient and age-matched control donor into the mitochondrial DNA (mtDNA) free human neuroblastoma (SH-SY5Y) cells [16]. The cybrid cells obtained from the University of Virginia. The resulting cell lines differed only in the source of mtDNA that repopulated the cells, but otherwise had identical nuclear genetic and environmental backgrounds, allowing for the in vitro elucidation of mitochondrial genomic differences [17].

### In vitro hypoxia and simvastatin treatment

Cultures were maintained in Dulbecco’s Modified Eagle’s Medium (DMEM) supplemented with 10 % fetal bovine serum (FBS), 100 U penicillin, and 0.1 mg/mL streptomycin at 37 °C under 5 % CO_2_/95 % O_2_ until reaching 70 % confluence. After starving the cells with DMEM containing 0.2 % FBS for 24 h, the cultures were placed in normoxic or hypoxic conditions with 1 μM or 10 μM simvastatin throughout the course of the experiments (0–12 h) [15]. Simvastatin was obtained from Chong Kun Dang Pharmaceutical Co., Ltd. (Seoul, South Korea). Treatments were performed in triplicate, and experiments were repeated three times.

All hypoxic ischemia experiments were performed with cultures incubated in a humidified hypoxic chamber. To induce hypoxia, the cultures were incubated in 93 % N_2_/5 % CO_2_/2 % O_2_ at 37 °C.

### Cell viability assay

Cell viability was determined by MTT (3-[4,5-dimethylthiazol-2-yl]-2,5-diphenyl tetrazolium bromide) assay. A stock solution of MTT (5 mg/mL in phosphate-buffered saline, pH 7.4) was freshly prepared, and the cells were incubated for 4 h at a final concentration of 1 mg/mL. The samples on each plate were read on an ELISA reader with a reference wavelength of 570 nm. The results are expressed as a percentage of absorbance at 490 nm directly proportional to the number of living cells following experimental hypoxia.

### HIF and BACE immunoassay

For immunoblot analysis, cells cultured on 100 mm plates were washed with 4 °C phosphate-buffered saline (PBS) and collected by centrifugation. The cells were homogenized in lysis buffer [100 mmol/L NaCl, 10 mmol/L Tris (pH 7.5), 1 mmol/L EDTA] with freshly prepared protease inhibitors (1 mM phenylmethylsulfonyl fluoride). Protein concentrations were determined using the Bradford method (Bio-Rad, Richmond, CA). Protein extracts (40 μg) were separated by 10 % sodium dodecyl sulfate-polyacrylamide gel electrophoresis and transferred to a nitrocellulose membrane. The membranes were blocked in 5 % nonfat skim milk in TBS (0.15 M NaCl, 25 mM Tris–HCl, 25 mM Tris) for 2 h, and then incubated at a 1:500 dilution overnight at 4 °C with anti-BACE (rabbit polyclonal antibodies, Millipore corporation) or anti-HIF-1α (mouse monoclonal antibodies, BD bioscience) antibodies. After washing 3 times in TBST (TBS + 0.5 % Tween-20), the membrane was incubated with secondary antibody (anti-rabbit or anti-mouse) for 1 h at room temperature. Immunoreactive bands were detected by enhanced chemiluminescence with Kodak film. All experiments were repeated three times.

### Statistical analysis

Graphical data represent the means (± SE) of at least three independent experiments. Luminograms are representative of at least three experiments with similar results. Statistical analysis was performed by Student’s *t*-test when appropriate. A P value of 0.05 was considered statistically significant in all cases.

### Ethics

All the experiments were conducted at Seoul National University Bundang Hospital and the study was approved by the local ethics committee of the Seoul National University Bundang Hospital.

## Results

### Simvastatin did not reduce hypoxia- induced cell deaths

**Fig. 1:**
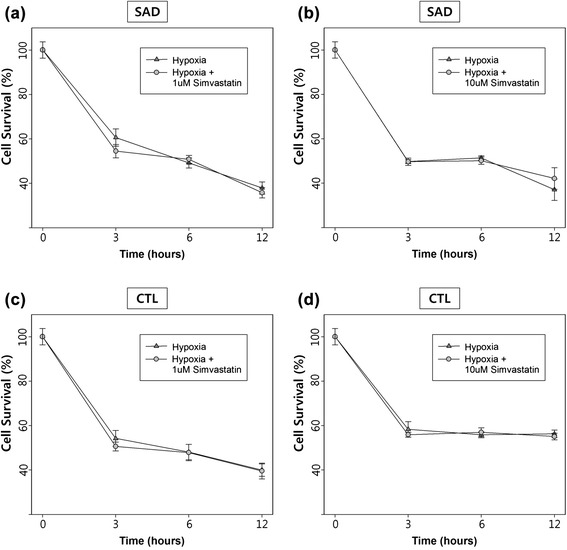
The effect of simvastatin on cell survival. Cells were incubated with 1 or 10 μM simvastatin under hypoxia. Cell viability was measured by MTT assay. (**a**, **b**, **c**, **d**) Cell survival decreased over time. There were no significant difference between cell survival in both SAD and CTL cybrids (*p* > 0.05). X-axis represents hypoxia duration; Y-axis represents mean percentage (± SE) of cell survival comparing to the 0 time point. All experiments were repeated three times

We analyzed the effect of simvastatin on cell viability under hypoxia. CTL and SAD cells showed reduced cell viability over 12 h hypoxic conditions (2 % O_2_). Between 0 h and 3 h, viability was reduced to 50 % of the control level, and after 12 h, 70 % of the cells were dead. After treatment with simvastatin (1 μM and 10 μM), there was no difference in survival between the SAD and CTL cybrids (Fig. 1).

### Low-dose simvastatin decreased HIF-1α and BACE expression in SAD cybrids

**Fig. 2:**
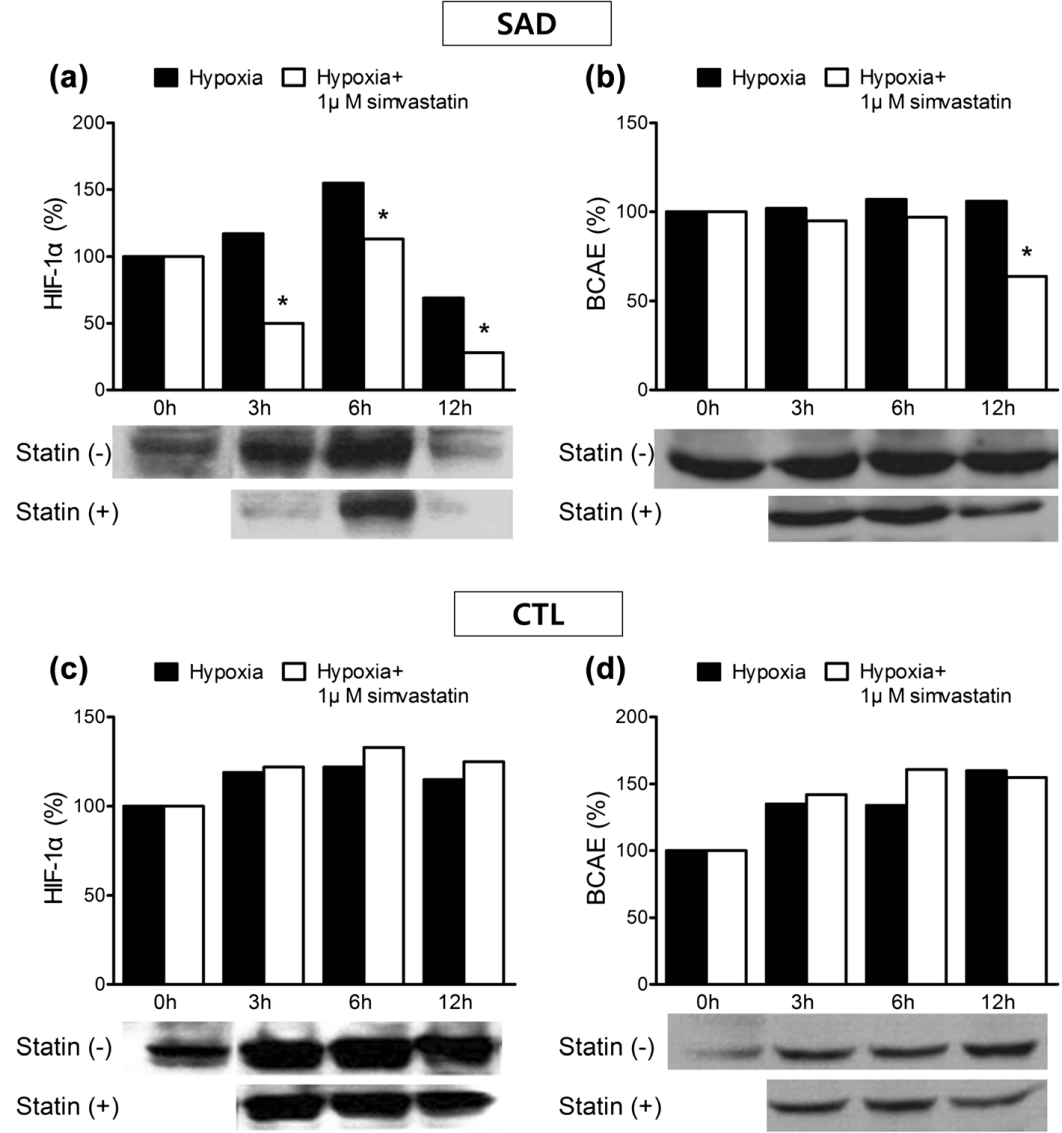
The effect of low-dose simvastatin on HIF-1α and BACE expression. Cells were incubated with 0 and 1 μM simvastatin under hypoxia. Intracellular HIF-1α and BACE levels were measured by western blotting. **a** In SAD cybrids, HIF-1α was significantly decreased at 3 h, 6 h, and 12 h in the presence of 1 μM simvastatin (*P < 0.05). **b** In SAD cybrids, BACE significantly decreased at 12 h in the presence of 1 μM simvastatin (*P < 0.05). **c**, **d** In CTL cybrids, 1 μM simvastatin did not influence HIF-1α and BACE expression. X-axis represents hypoxia duration; Y-axis represents percentage value from the immunoassay versus the 0 time point. All experiments were repeated three times

**Fig. 3:**
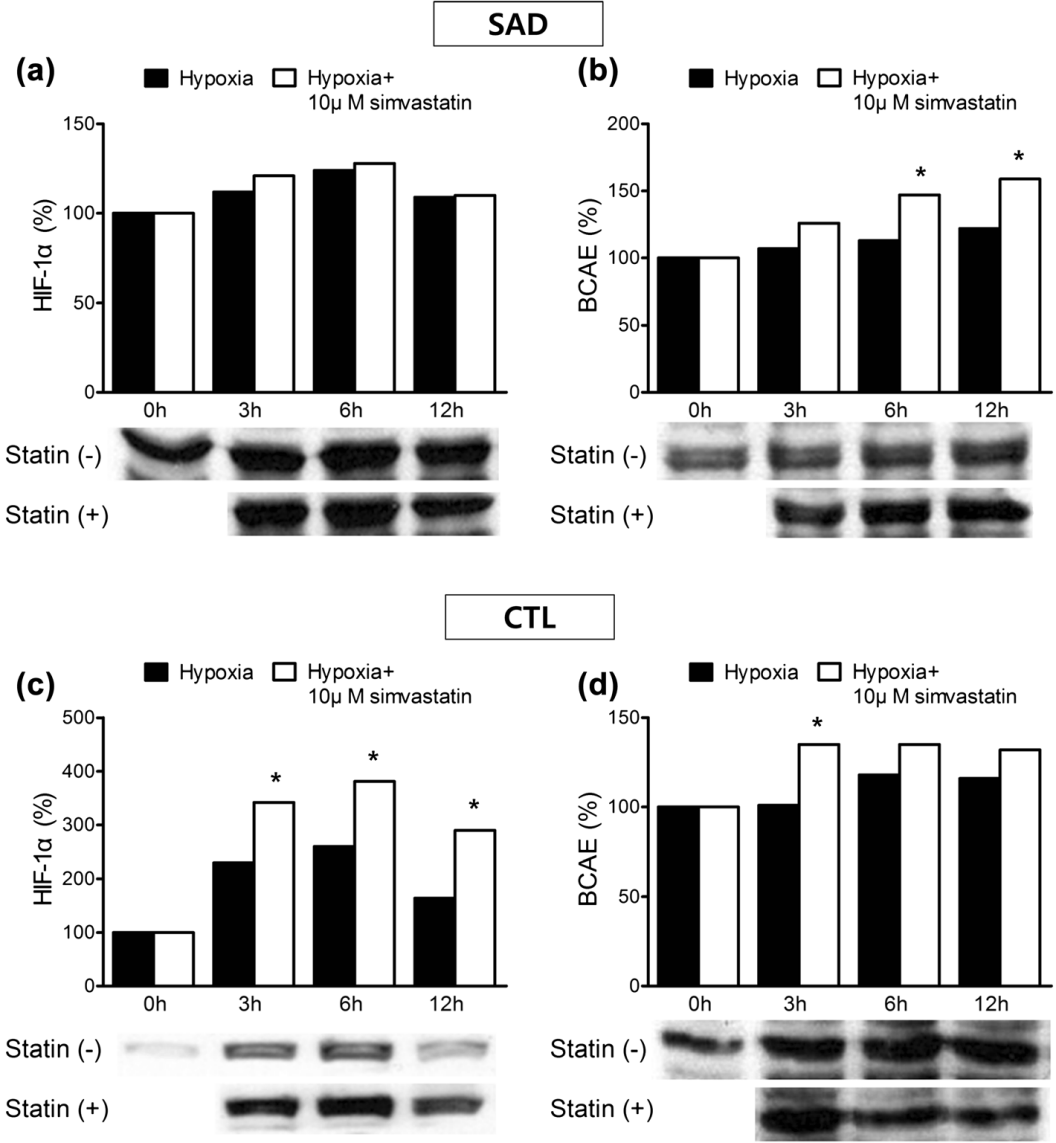
The effect of high-dose simvastatin on expression of HIF-1α and BACE. Cells were incubated with 0 and 10 μM simvastatin under hypoxia. Intracellular HIF-1α and BACE levels were measured by western blotting. **a** In SAD cybrids, 10 μM simvastatin did not influence HIF-1α. **b** In SAD cybrids, BACE significantly increased at 6 h and 12 h in the presence of 10 μM simvastatin (*P < 0.05). **c** In CTL, HIF-1α significantly increased at 3 h, 6 h, and 12 h in the presence of 10 μM simvastatin (*P < 0.05). **d** In CTL, BACE significantly increased at 3 h in the presence of 10 μM simvastatin (*P < 0.05). X-axis represents hypoxia duration; Y-axis represents represents percent values from the immunoassay versus the 0 time point. All experiments were repeated three times

In order to determine the effect of simvastatin on HIF-1α mediated BACE expression, we used immunoassay. Hypoxia increased expression of HIF-1α and BACE in both CTL and SAD cybrids (Figs. 2 and 3). HIF-1α levels increased rapidly for the first 6 h, and began to decrease at 12 h. BACE levels gradually increased throughout the 12 h period. After treatment with 1 μM simvastatin, HIF-1α and BACE levels decreased in the SAD cybrids (Fig. 2a, 2b). HIF-1α levels decreased by 70 % (3 h), 40 % (6 h), and 40 % (12 h) with 1 μM simvastatin (*P < 0.05) (Fig. 2a). BACE levels decreased at 12 h (40 %), but there was little change at 3 h (<10 %) and 6 h (10 %) (Fig. 2b). The reduction in HIF-1α expression was prominent at 3 h, and the reduction in BACE expression was pronounced at 12 h.

In CTL cybrids, treatment with 1 μM simvastatin did not significantly affect HIF-1α and BACE expression (Fig. 2c, 2d).

### High-dose simvastatin increased HIF-1α and BACE expression in SAD and CTL cybrids

Treatment with 10 μM simvastatin increased expression of HIF-1α and BACE (Fig. 3). HIF-1α levels increased by up to 10 % (3–12 h) in SAD cybrids and 110–130 % (3–12 h) in CTL cybrids. The increase in HIF-1α expression was significant at 6 h and 12 h in CTL cybrids, but the change was smaller in SAD cybrids. BACE levels increased by 20–40 % (3–12 h) in SAD cybrids, and 20–40 % (3–12 h) in CTL cybrids. BACE expression significantly increased at 6 h and 12 h in SAD cybrids, and at 3 h in CTL cybrids (*p < 0.05).

## Discussion

This study investigated the changes in HIF-1α and BACE levels in the presence of simvastatin under hypoxic conditions in AD cybrid cells. In SAD cybrids, HIF-1α and BACE levels decreased by 40–70 % with low-dose simvastatin; however, high-dose simvastatin increased the expression of HIF-1α and BACE up to 130 %. Aβ is derived from β-amyloid precursor protein by proteolytic cleavage from β-secretase via induction of BACE [4–6]. Increased BACE activity and elevated insoluble Aβ peptide have been shown in brain tissue of patients with AD, suggesting that abnormal BACE activity contributes to AD pathogenesis [5]. Stroke or ischemia gives rise to hypoxic conditions known to increase the incidence of AD and hypoxia increases transcription of BACE via overexpression of HIF-1α [3, 5]. In this study, low-dose simvastatin reduced HIF-1α mediated BACE production from hypoxic injury in SAD cybrids, but not in high-dose.

Other studies have shown the dose-dependent effects of statin on biochemical markers. High-dose simvastatin (10 μM) increased Aβ release from HEK cells, but low-dose simvastatin (1 μM) showed little difference [15]. One study demonstrated the dose-dependent effect of atorvastatin on endothelial cell migration and angiogenesis [18]. Low-dose statin promotes migration of mature endothelial cells and progenitor cells that contribute to vasculogenesis. However, high-dose statins block angiogenesis and migration by inducing endothelial cell apoptosis. In cortical neuronal cells, low-dose simvastatin (100 nmol) protects against cytotoxcity by enhancing expression of Bcl-2 mRNA [19]. Pre-incubation with low-dose simvastatin reduces Aβ peptide-induced cell death in cortical and cerebellar neurons [20]. If in vitro experiments correctly reflect pathophysiological events that take place in the human brain, chronic low-dose statin administration may be therapeutically beneficial.

Cholesterol is an important factor in the regulation of Aβ production. High-dose statins inhibit cholesterol synthesis, and low cellular cholesterol levels reduce Aβ secretion [13, 14]. Low-dose statins preferentially inhibit isoprenoid biosynthesis [21, 22] and inhibition of β-secretase dimerization by low isoprenoid reduces Aβ production [15, 23]. Statins also modulate the DNA-binding activity of HIF-1α and simvastatin attenuates HIF-1α expression in vascular smooth muscle cells [10, 23].

Several groups have reported the benefit of statins in the treatment of AD. In patients with mild AD, high-dose simvastatin (80 mg/day) treatment reduced Aβ levels in the cerebrospinal fluid (CSF) [24]. In addition, 20 mg simvastatin reduced CSF levels of amyloid precursor protein in patients with AD [25]. Lovastatin (10–60 mg/day) produced a dose-dependent decrease in serum Aβ in patients with elevated levels of low-density lipoprotein cholesterol [26]. These studies showed the benefit of statin treatment in reducing Aβ production in humans, but differences between the types and doses of statin have not been satisfactorily defined.

Patients with AD are susceptible to chronic hypoxia [2, 27]. Disrupted perfusion is present in the early phases of AD [2]; consequently, a reduction of oxygen delivery to the brain promotes mitochondrial dysfunction and apoptosis [17]. As expected, SAD cybrids derived from AD patients are also susceptible to chronic hypoxia. We used a cybrid cell model to evaluate mitochondrial dysfunction in AD patients under hypoxic conditions. Mitochondrial dysfunction in the presence of oxidative stress is intimately involved with AD pathophysiology. The mitochondrial electron chain acts as an oxygen sensor, releasing reactive oxygen species in response to hypoxia, thereby promoting oxidative stress, leading to cell death [28]. Mitochondrial dysfunction is observed in the platelets and lymphocytes of AD patients and their postmortem brain tissue [29].

Several studies have shown that statin activity on Aβ production is mediated by BACE [15, 22, 30]. In vitro and in vivo studies have shown that overexpression of BACE elevates Aβ production [31, 32]. This up-regulation of BACE and hypoxic stress are thought to have pathogenic relevance to neurodegeneration and dementia. Although we did not directly measure Aβ, BACE activity is estimated as an indirect biological marker of Aβ production.

## Conclusions

This study was designed to determine the effects of simvastatin on the expression of HIF-1α and BACE in cybrid cells as possible important mediators of amyloid precursor protein processing. We demonstrated a dose-dependent differential response of simvastatin on HIF-1α and BACE expression. While low-dose simvastatin reduced the expression of HIF-1α and BACE under hypoxia in SAD cybrids, high-dose simvastatin increased the expression of both markers. Thus, our studies suggest the potential utility of therapeutic low-dose simvastatin regimen to control HIF-1α and BACE expression, which may prevent beta amyloid production. Additional translational studies in transgenic mice are required to demonstrate beneficial effects of simvastatin on amyloid load and reversal of behavioral deficits prior to clinical studies in AD patients.

## Abbreviations

AD: Alzheimer’s disease
Aβ: beta amyloid
BACE: β-site amyloid precursor protein cleaving enzyme
CTL: age matched controls
DMEM: Dulbecco’s Modified Eagle’s Medium
FBS: fetal bovine serum
HIF-1α: hypoxia inducible factor 1α
mtDNA: mitochondrial DNA
MTT: 3-[4,5-dimethylthiazol-2-yl]-2,5-diphenyl tetrazolium bromide
PBS: phosphate-buffered saline
SAD: sporadic Alzheimer’s disease.
